# Band Selection for Dehazing Algorithms Applied to Hyperspectral Images in the Visible Range

**DOI:** 10.3390/s21175935

**Published:** 2021-09-03

**Authors:** Sol Fernández-Carvelo, Miguel Ángel Martínez-Domingo, Eva M. Valero, Javier Romero, Juan Luis Nieves, Javier Hernández-Andrés

**Affiliations:** Department of Optics, University of Granada, 18071 Granada, Spain; solfcarvelo@ugr.es (S.F.-C.); martinezm@ugr.es (M.Á.M.-D.); jromero@ugr.es (J.R.); jnieves@ugr.es (J.L.N.); javierha@ugr.es (J.H.-A.)

**Keywords:** dehazing, hyperspectral, band selection, image quality metric

## Abstract

Images captured under bad weather conditions (e.g., fog, haze, mist, dust, etc.), suffer from poor contrast and visibility, and color distortions. The severity of this degradation depends on the distance, the density of the atmospheric particles and the wavelength. We analyzed eight single image dehazing algorithms representative of different strategies and originally developed for RGB images, over a database of hazy spectral images in the visible range. We carried out a brute force search to find the optimum three wavelengths according to a new combined image quality metric. The optimal triplet of monochromatic bands depends on the dehazing algorithm used and, in most cases, the different bands are quite close to each other. According to our proposed combined metric, the best method is the artificial multiple exposure image fusion (AMEF). If all wavelengths within the range 450–720 nm are used to build a sRGB renderization of the imagaes, the two best-performing methods are AMEF and the contrast limited adaptive histogram equalization (CLAHE), with very similar quality of the dehazed images. Our results show that the performance of the algorithms critically depends on the signal balance and the information present in the three channels of the input image. The capture time can be considerably shortened, and the capture device simplified by using a triplet of bands instead of the full wavelength range for dehazing purposes, although the selection of the bands must be performed specifically for a given algorithm.

## 1. Introduction

In outdoor conditions, captured images are affected by the absorption and scattering of particles in the atmosphere as they travel from the scene to the camera [1,2], reducing the contrast and visibility of the objects and changing their color [3]. This degradation depends on the distance, the density of the atmospheric particles and the wavelength. We can differentiate between haze and fog by taking into account not only the radius size of water particles (less than 1 micron corresponds to haze) but also their concentration (more than 100 particles/cm^3^ is considered as fog) [4,5]. In either case, these factors make it difficult to identify the main features of the objects recorded in the image, especially in distant scenes with high haze and/or fog density, which hinders further image-processing tasks due to poor visibility, whether performed by a human observer or by computer vision algorithms.

Dehazing (or defogging) techniques aim to eliminate or reduce this degradation and improve the visual quality of images for computer-aided applications and human interpretation. The objective of these methods is to obtain an image as free as possible from either haze or fog. Image dehazing is an indispensable step in many applications such as air and maritime transport, surveillance, driver assistance systems, remote sensing, agronomy, archaeology, astronomy, medical sciences and environmental studies [6,7,8,9,10,11,12,13,14,15,16,17,18,19]. In the last decade, a large number of dehazing algorithms have been proposed, and this has become a growing area of research and development. Depending on the proposed paradigm, dehazing methods can be classified in different ways [5,20,21]. Thus, for example, it is usual to distinguish between methods based on image enhancement [22,23,24,25,26], methods based on image fusion [27,28], methods based on image restoration [29] and Deep-Learning based methods, which often combine different strategies in the network design [30,31,32,33]. In addition, we can find classifications that differentiate between methods based on physical models [34] and methods based on the statistical information contained in each scene (not based on physical models) [35,36,37]. On the other hand, we can find a third classification based on the number of images used: a single image or multiple images [38].

The most recent dehazing techniques mainly focus on the single image strategy. Such techniques have gained popularity due to the Dark Channel Prior (DCP) method proposed by He et al. [39]. Although physical model-based methods provide more accurate image retrieval, they fail to produce good results when the haze present in the scene is not well modeled [40,41]. Such physical model-based methods are based on Koshmieder’s law [4,29] (see Equation (1)). This is a pixel-to-pixel wavelength-dependent dichromatic atmospheric scattering model that combines the direct transmission and airlight terms to describe the spectral radiance of the image I(x, λ) as

(1) $$I\left(x,\lambda \right)=L_{0}\left(x,\lambda \right)e^{-\beta \left(\lambda \right)d\left(x\right)}+L_{\infty }\left(x,\lambda \right)\left(1-e^{-\beta \left(\lambda \right)d\left(x\right)}\right),$$

where x refers to the 2D pixel coordinates in the image (x_1_,x_2_), I(x, λ) represents the spectral radiance of the captured image, L_0_(x, λ) is the spectral radiance coming from the spatial location x (i.e., the object), L_∞_ (x, λ) is the horizon spectral radiance, β(λ) is the atmospheric extinction coefficient, λ the wavelength and d(x) the distance between the object and the imaging device. As discussed above, the light reflected by the objects is attenuated, which can be spectrally selective, due to scattering and absorption phenomena along the path from the objects to the observer. In addition, light scattered in the atmosphere by particles beyond this path is added to the direct light. This is the component of the light received by the observer, called airlight (A(x)) and defined in Equation (2) [42]. The transmission (t(x)), which describes the portion of the light that is not scattered and therefore reaches the camera, is defined as shown in Equation (3). Regrouping the airlight (A(x)) and the transmission terms (t(x)) such that as

(2) $$A\left(x,\lambda \right)={\;L}_{\infty }\left(x,\lambda \right)\left(1-e^{-\beta \left(\lambda \right)d\left(x\right)}\right),$$

(3) $$t\left(x,\lambda \right)=e^{-\beta \left(\lambda \right)d\left(x\right)},$$

the above Equation (1) can be rewritten as Equation (4).

(4) $$I\left(x,\lambda \right)=D\left(x,\lambda \right)+A\left(x,\lambda \right),$$

where $D\left(x,\lambda \right)=L_{0}\left(x,\lambda \right)t\left(x,\lambda \right)$ represents the direct transmission. 

In most single-image dehazing procedures found in the literature, the main drawback of using the above law is that the number of unknowns is larger than the number of equations. In general, β depends on the wavelength and it is different for each color channel. To solve this problem, various dehazing methods often assume that the dependence of β with wavelength is negligible for the estimation of both airlight and transmission from a single RGB image with haze [43].

Thanks to advances in image sensors and spectral filters, interest in multispectral and hyperspectral imaging systems has increased in recent years and the range of application fields has widened considerably [5]. Despite this, in general there is a great scarcity of hyperspectral imaging databases in the domain of image dehazing, especially in uncontrolled conditions.

In a previous study [5], we investigated how dehazing methods behave in the hyperspectral imaging domain. Our main aim was to test whether dehazing algorithms, originally developed to work on RGB images, were able to deal with spectral images and if they would perform better for particular wavelengths. To tackle this objective, an adaptation of several single-image dehazing methods was performed by constructing a three-channel monochrome image (with the same information in each channel, corresponding to one wavelength). Subsequently, we evaluated them based on the analysis of eleven state-of-the-art metrics. 

Since most of the existing dehazing methods work with three channel color images as input, in this study we have taken a step forward by searching for the optimal triplet of spectral bands to use as input for a given algorithm. This can also be related to the simplification of the capture device used for a particular application. The selection of the three optimum bands has been carried out by means of a brute force search strategy imposing some initial constraints for better optimization. We also carried out the performance evaluation of eight dehazing algorithms in a different way than we used in the previous study [5]. In this work, we introduce a new combined metric of image quality evaluation using three metrics commonly used in dehazing [44,45,46]. We also studied the performance of the different algorithms using the information from all wavelengths within the range 450–720 nm to produce a sRGB renderization of the spectral image. The main contributions of this paper are: the selection of the three optimum bands in the visible range by an exhaustive search method, from the original hyperspectral images which contained 28 bands; the evaluation of eight state-of-the-art single-image dehazing algorithms and the proposal of a combined metric for image quality evaluation, built from three image quality metrics that take into account different aspects of evaluating contrast enhancement and the presence of artifacts in the image.

The paper is organized as follows: Section 2 describes the database used, Section 3 the selected dehazing methods, Section 4 the image quality metrics used for performance evaluation, Section 5 both the procedure followed to obtain the optimal bands and the renderization method, Section 6 shows the analysis of the results obtained and the comparison between the two strategies. Finally, Section 7 discusses the conclusions and possible objectives for future work.

## 2. Spectral Hazy Image Database

Currently, a number of useful databases are available for use in dehazing, but the specific characteristics of each database can significantly influence the expected results. Most of them are composed of conventional color images or a combination of color and infrared images. It is important to note that many of them are databases without a reference image (i.e., a haze-free image of the same scene). This is due to the difficulty encountered in capturing the same scenes with identical conditions, save for the presence of haze. For this reason, the need to generate synthetic hazy images by computation and artificial hazy images using haze machines has increased. A brief review of the currently available databases can be grouped into three categories: databases of real haze-affected images [47,48,49,50], databases of simulated haze-affected images [6,51,52,53,54,55,56,57] and databases of artificially generated haze-affected images [58,59,60,61,62,63,64]. To the best of our knowledge, there is only one database for dehazing that contains spectral images (captured in controlled conditions), called the SHIA (Spectral Hazy Image Database) [64]. Here, we used this database [64], composed of real image pairs captured with and without various haze densities under the same conditions. The details about the capture device can be found in [64]. The SHIA database has two scenes and ten density levels of artificially generated haze whose particles are water droplets with a radius close to that of the atmospheric fog (1–10 µm). We selected the M1 scene from SHIA database, which is in the 450–720 nm spectral range with a spectral resolution of 10 nm and a medium haze density level (level 7). The image size is 1312 × 1082 pixels and the integration time is 530 ms, for all wavelengths. The fact that the integration time is the same for all wavelengths in this database [64] results in an increase of noise for short wavelengths due to the low spectral response to the irradiance incident on the sensor. The low spectral response at wavelengths below 500 nm is caused by the low responsivity of CMOS and the low transmittance of the liquid crystal tunable filter, which are not uniform throughout the spectrum and results on lower values for short wavelengths within the visible range [17].

## 3. Dehazing Methods

We selected the following eight state-of-the-art dehazing algorithms for this study: Dark Channel Prior (DCP) [39], Meng [65], DehazeNet [66], Berman [43], Contrast Limited Adaptive Histogram Equalization (CLAHE) [67], RGB response ratio constancy (Luzón) [68], Artificial Multiple Exposure image Fusion (AMEF) [69] and Image Dehazing and Exposure Using an Enhanced Atmospheric Scattering Model (IDE) [26]. These are a set of algorithms representative of different strategies within the single image dehazing approach.

### 3.1. The Dark Channel Prior (DCP) Method

This method [39] is based on the statistics of outdoor haze-free images. It is based on the assumption that, in most local windows of a color image, at least one-color channel contains very low intensity pixels. This channel is called the dark channel and these pixels directly provide a robust estimate of the haze transmission. By adding a smoothing step, it is possible to create a depth map and recover high quality haze-free images. This is a method capable of handling distant objects in images with high haze density. One of its drawbacks is that due to its use of statistical information, it may not work well for certain images, such as those in which the objects in the scene are very similar to the airlight and no shadows are cast on them. In addition to this, as it is based on the dichromatic atmospheric scattering model, it may not work well in certain situations: it assumes constant airlight (when the sunlight is very influential this assumption is not adequate) and constant transmission for all color channels (when atmospheric particles are very small or objects are very far away the transmission depends on the wavelength and would be different for each color channel). Moreover, a good choice of the window size of the selected image is important; if it is too large, halos appear near edges and if it is too small we would have an oversaturated image (although it is assumed that the larger the window size, the more likely we are to find a darker pixel). On the other hand, resizing the input image might be needed due to its slow computation time, and it tends to produce a high increase in contrast. This introduces an alteration in the appearance of the image that is more noticeable for long wavelengths and low haze density [5]. In spite of this, it is important to mention that the alteration of the image appearance is not necessarily a limitation; it depends very much on the intended application. When the aim is to obtain a better contrast or to make the objects present in the scene more visible for object or text recognition, for example, this fact would not be a disadvantage).

### 3.2. The Meng Method

An often-overlooked point that has already been cited is the fact that single-image-based methods present a problem due to the sparsity of constraints. In this method [65], this aspect is improved by modifying the Dark Channel Prior concept [39]. This is carried out by introducing a set of lower bound constraints followed by a contextual regularization for transmission estimation that can better handle bright sky regions. It provides comparable results to Dark Channel Prior in situations with high haze density, although Meng’s method performs better in regions of non-uniform depth in the scene. However, it neglects the dependence of airlight with wavelength and it produces artifacts in the presence of rather large and achromatic objects.

### 3.3. The DehazeNet Method

An important aspect in the improvement of dehazing is the estimation of the transmission map for a hazy image. In this method [66], this estimation is performed using a trainable convolutional neural network system. The neural network is trained with synthetic hazy images using a simplified model for transmission estimation. The naturalness is maintained, without increasing the contrast too much [5]. Although an improvement in training is needed for a more accurate estimation of the transmission map, DehazeNet is able to locate the region corresponding to the sky (if present in the scene), keeping its color and ensuring an adequate haze removal in other regions. DehazeNet also has the limitation of assuming the airlight term is a constant, but the estimation of airlight and transmission is better than the one provided by DCP [39]. The main drawback is the lack of success at high haze densities (due to the low haze density present in the images used for training). Although there are other methods based on Deep Learning [30,31,32,33,70,71], we chose this one as a representative of the Deep Learning strategy because it is the most widely used in the literature.

### 3.4. The Berman Method

The degradation effect due to haze is different for each pixel of the image and depends on the distance between the object location and the imaging device. This method [43] proposes a pixel-to-pixel transmission estimation. It is assumed that the colors of a haze-free image can be approximated by using a series of colors from different distances that form groups in the RGB space (called haze lines). This method needs to apply a radiometric correction to obtain pixels with homogeneous intensity values, as it can fail when the airlight is brighter than the objects in the scene. In addition, the results show a degradation of performance with increasing noise and some alteration in terms of naturalness, with increased contrast [5].

### 3.5. The CLAHE Method

In an attempt to address the problem of low contrast in images affected by haze, a limited adaptive histogram equalization method of contrast enhancement is proposed in [67]. It is an improvement on previously developed histogram equalization methods [72] (one of the most successful non-physical model-based strategies). This is achieved by converting the image to the HSI color space, imposing a maximum value for each pixel intensity and cropping the histogram, redistributing the cropped pixels evenly across each gray level. One important aspect is that it is not designed for dehazing, so it is not based on the dichromatic atmospheric scattering model and it works correctly for removing haze with a constant thickness. On the other hand, it introduces some noise in the dehazed image that increases with haze density [64]. Its main advantages are its short processing time and its simplicity.

### 3.6. The Luzón Method

This method has been developed based on the results previously obtained by different authors [68] that found a linear relationship with a high correlation coefficient between the pairs of excitation values of each cone of the human visual system (L, M and S) for different objects under different illuminants [73]. The method is based on the constancy of the response ratio of the sensors of a RGB camera when objects are observed under two different illuminants (haze-free vs. hazy images in a dehazing context). Its purpose is to achieve an image with a low haze component and objects with more contrast. Satisfactory results in terms of visibility, contrast and color are achieved in the restored images without prior knowledge of the image characteristics. As in CLAHE [67], its simplicity, speed of processing and good color recovery performance are relevant aspects of this method. However, it does not deal well with very distant objects and high haze densities. 

### 3.7. The AMEF Method

The greater the depth in the image scene, the greater the degradation caused by haze. Methods that start from a single image are intended to achieve enhancements without external knowledge. However, even simplified physical models need information about object distances in the image to obtain satisfactory contrast enhancement or color recovery. Galdran [69] proposed a new approach for dehazing called Artificial Multi-Exposure Fusion (AMEF), without the need of prior information about distance, and partially based on physical models. An artificial modification of the exposure is performed by gamma correction, generating an additional set of both underexposed and overexposed images. This is followed by a fusion of the multi-exposed images. Then, the underexposed images are used for obtaining the haze map. Finally, CLAHE [67] is applied for further contrast enhancement. The main advantage of this method is its speed, and that it avoids the need to estimate the airlight and transmission map. Its main weakness is that the haze thickness must be constant to obtain satisfactory results. 

### 3.8. The IDE Method

The atmospheric scattering model is widely used as a base for image dehazing algorithms. However, it is fair to point out that this model has an intrinsic limitation, which leads to a dim effect in the recovered results. The limitation is related to the absorption of light, which takes place in the scene and it is influenced by local factors (depth, texture of the objects present in the scene). A reformulation of the atmospheric scattering model including several additional parameters was recently proposed by Ju et al. (2021) and included in a dehazing model based on image processing within local image patches, called Image Dehazing and Exposure (IDE) [26]. Relying on this enhanced atmospheric scattering model, a simple yet effective gray-world-assumption-based technique is used to enhance the visibility of hazy images, with high quality results. It is worth mentioning that IDE does not require any training process or extra information related to scene depth, which makes it very fast and robust. Moreover, the stretching strategy used in IDE can effectively avoid some undesirable effects in the dehazed images, e.g., over-enhancement, over-saturation, and mist residue, among others [26].

## 4. Image Quality Metrics

Many image quality assessment methods have been used for the evaluation of dehazing algorithms in the literature [20]. They can be roughly classified into three categories: full-reference metrics that require the haze-free image as reference [44,45,46,74,75], reduced reference metrics that use the hazy image as reference [76,77,78] and no reference metrics [79,80]. Using full reference metrics requires databases that include corresponding haze-free images, which are not very common as we commented in Section 1. Moreover, the fact that each metric evaluates different image features or characteristics must be considered. Therefore, it is necessary to establish common ground to choose an adequate set of metrics for the analysis of results in our experiments. 

We worked with three-channel images in this study, as described in Section 1. The method for generating these images is fully detailed in Section 5. Since we used a database that contains reference haze-free images, we chose a subset of full-reference metrics for the analysis of our results. The selected metrics are Peak Signal-to-Noise Ratio (PSNR) [81], multi-scale structural similarity (MS-SSIM) [44], visual information fidelity (VIF) [45] and multi-scale improved color image difference (MS-iCID) [46].

### 4.1. Peak Signal-to-Noise Ratio (PSNR)

This metric [81] is related to MSE (mean square error) [82], which directly compares two images through the mean of the pixel-by-pixel squared differences between the two images. Both PSNR and MSE are complementary metrics. Since PSNR is inversely proportional to MSE, the higher the value of PSNR, the more similar two images are. It makes sense to use this metric for comparison between the restored image and the ground truth (haze-free) image. Some care needs to be taken when interpreting the metric’s results, because a low PSNR value could be due to mean gray level or contrast differences. It belongs to the group of full-reference metrics.

### 4.2. Multi-Scale Structural Similarity (MS-SSIM)

The well-known SSIM image quality metric [83] exploits the fact that images have structured signals that are dependent on neighborhood pixels [84], and that the human visual system is used to extract structural information from a given scene. SSIM tackles image quality evaluation by being sensitive to distortions that are critical for a human observer’s perception of the scene. Three components are considered in this metric: luminance, contrast and structure. The multi-scale structural similarity metric (MS-SSIM) was introduced as an improvement to SSIM that considers the best spatial scale according to the visualization conditions of the scene (viewing distance and display resolution). So MS-SSIM integrates different spatial resolutions and viewing distances into its definition to take this factor into account [85]. The closer the metric value to unity, the more similar the two images being compared are.

### 4.3. Visual Information Fidelity (VIF)

VIF [45] evaluates the quality of the dehazed image based on an estimation of the amount of shared information between the haze-free and dehazed images. The loss of information due to distortions is quantified using natural scene statistics (NSS), a simplified human visual system (HVS) model and an image distortion model in an information-theoretic framework. The reference image is modeled as being the output of a stochastic ‘natural’ source that passes through the HVS channel and is later processed by the brain. Then, the information content of the reference image is quantified as being the mutual information between the input and output of the HVS channel. This is the information that the brain could ideally extract from the output of the HVS. Finally, the amount of information of a distorted image is quantified, thereby estimating the information that the brain could ideally extract from the test image. VIF is then calculated as the ratio between the two information estimations corresponding to the test and reference images. The closer to unity the value is, the better the result, although the metric value can also be above unity if an enhanced version of the image is compared with the original version.

### 4.4. Multi-Scale Improved Color Image Difference (MS-iCID)

This metric was proposed [46] as an improvement of the color image difference metric (CID) [86], which is the version of SSIM adapted to color difference assessment. As with CID, hue and chroma differences are considered apart from the three factors originally used in SSIM, but also multiple scales are applied to the image to obtain the final result. This metric can be used for distortion assessment in gamut mapping applications [41]. The closer the value of the result to zero, the more similar the two images being compared are. 

### 4.5. Combined Metric for Dehazed Image Evaluation (CM-DIE)

As described in Section 1, in this study we proposed a novel metric for quality assessment of dehazed images known as the combined metric for dehazed image evaluation (CM-DIE), which is a combination of the MS-SSIM [44], VIF [45] and MS-iCID [46] metrics. This combined metric, CM-DIE, yields a unique value for image quality assessment, which allows for the brute-force optimization method to be carried out for selecting the best three bands for each dehazing algorithm since the results depend on the metric used taking into account that different features are evaluated. The combined metric is defined as shown in Equation (5):

(5) $$C\mathrm{M-}DIE=W_{1}\;\left|1-VIF\right|+W_{2}\left(MSiCID\right)+W_{3}\left(1-MSSSIM\right),$$

where, *W*_1_ = 0.146, *W*_2_ = 0.309 and *W*_3_ = 0.545 are relative weights, *VIF* represents the VIF value, *MSiCID* the MS-iCID value, *MSSIM* the MS-SSIM value for the image pair, and the straight bars represent the absolute value function. The relative weights have been computed to ensure that each term of the metric has the same mean value in our set of 1540 images. The weights have been normalized to unity. As it is defined, the closer the metric value to zero, the more similar the two images being compared are.

## 5. Algorithm Parameter Selection and Brute Force Optimization

### 5.1. Algorithm Parameters

Most of the dehazing methods selected for this study have been designed to work with three-channel color images. Most of them are also parametric. We decided to use most parameters as set in the original references. A few of them have been modified to achieve the best results possible for each algorithm, as described below.

For the DCP [39], the constant parameter that controls the small amount of haze considered for the distant objects (ω) has been set to 0.95, patch size 15 × 15 pixels, the maximum width and height of the image is 1313 pixels and the neighborhood for the median filter is 5 × 5 pixels.

For the Meng method [65], there are many parameters that need to be fixed: the patch size for airlight estimation (12 pixels), the patch size for transmission restriction (3 pixels), the regularization parameter (λ = 2), the parameter serving as the role of the medium extinction coefficient (δ = 0.85), the transparency parameter (α = 0.5), and the radiance bounds (C0 = 30 and C1 = 300).

For the Berman method [43], the gamma correction value is set to 1, and the rest of the parameters are set as shown in the original reference. 

For the IDE algorithm, all parameters have been set as in the original reference [26], since we found that the quality of the dehazed image was not influenced significantly by changing the window size and percentage of pixels that are saturated in the image within reasonable limits.

### 5.2. Brute-Force Band Optimization

The SHIA Database [64] spectral range covers 28 channels between 450 nm and 720 nm. The aim of the band selection step is to find the optimum triplet of bands for the different dehazing methods, so we used a false color three-channel image as the input for the dehazing algorithms. We imposed several restrictions to the brute-force optimization, to initially reduce the number of combinations that were possible. We only considered combinations of three bands with the following restrictions: (1) minimum distance of 40 nm between each pair of bands; and (2) bands ranked from higher to lower wavelengths, emulating the RGB ranking of the channels in a conventional color image. With these restrictions, the possible combinations were reduced from 21952 to 1540, allowing us to perform the optimization via brute-force and thus test all the possible combinations. This method ensures that the optimal combination within the restricted group of three band images for our dataset is found. The optimal combination was the one corresponding to the minimum value of the CM-DIE.

## 6. Results and Discussion

In this section, we evaluate the results obtained for the different dehazing methods tested using the quantitative data of quality metrics. We also show (just for visualization purposes) the original haze-free, hazy and dehazed images in grayscale that correspond to the optimum three bands selected for each algorithm. To generate this grayscale image, we averaged the intensity values of the three bands, to allow for better comparison amongst methods with different optimum triplet bands.

**Figure 1:**
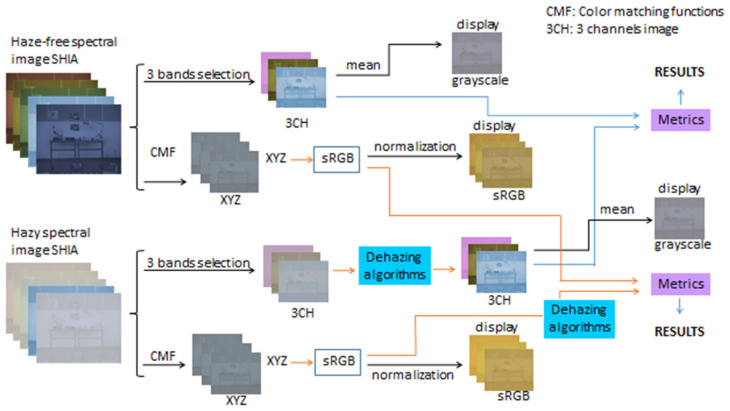
Schematic workflow of the study.

Finally, we used all wavelengths data within the range 450–720 nm to generate the CIE 1931 XYZ tristimulus values for each pixel of the scene, and we transformed these XYZ values to sRGB values using the standard transformation [87]. This allowed us to obtain the quality metrics corresponding to a standard sRGB color image generated from the spectral data, and compare the values with the quality obtained using the optimal triplets for each method. In Figure 1, we show a detailed workflow of the generation of the results and the analysis procedure.

### 6.1. Quality Metrics for The Optimum Triplet Band for Each Dehazing Algorithm

In Table 1, five different quality metrics (PSNR [81], MS-SSIM [44], VIF [45], MS-iCID [46] and CM-DIE) mean and standard deviation for the 1540 tested triplets are shown for each dehazing algorithm, along with the wavelength triplet bands selected by the brute force approach described in Section 5. The range of values between the minimum and the maximum obtained for the CM-DIE metric is also shown in the column named ‘Range’.

The best-performing algorithm is shown in blue, and the second best in green. Not all the metrics shown in Table 1 produce the same ranking of the algorithms, which is not surprising if we consider that they are sensitive to different image changes, or that they quantify these changes (artifacts or distortions) in different ways. Nevertheless, all of them, save for PSNR, rank AMEF as the best-performing method and CLAHE as the second best on average for the 1540 images. According to the PSNR metric values, DCP shows the worst result, indicating less similarity between the haze-free and dehazed images. The VIF values are above unity for DCP and CLAHE, which shows that the changes introduced by the dehazing algorithms could be consistent with image enhancement in some features such as contrast. The MS-SSIM shows less discriminative capacity (the best- and worst-performing algorithms are closer in value for this metric than for the others), and the worst result corresponds to the DCP method, in agreement with the PSNR results. For MS-iCID the worst values correspond to the IDE, DehazeNet and Luzón methods, maybe reflecting some alteration in the distribution of intensity values within the bands as a consequence of dehazing. We focused our analysis mainly on the values of the combined metric (CM-DIE), since the optimization was performed according to the distribution of values of this metric. The smallest value of standard deviation corresponds to CLAHE followed by AMEF, which is also related not only to a lesser dispersion of the values of the metric, but also to a smaller average value. The difference between AMEF and CLAHE is not very large for the average CM-DIE value, standard deviation or range. 

The worst-performing methods are DehazeNet, IDE and Luzón. In the case of DehazeNet, it is likely that the amount of haze in the images was too high for it to perform acceptably, a trend that we also found in a previous study with single band images [5]. Regarding Luzón, the very reduced distance range and the uniformity in haze thickness in the scene are generally unfavorable for methods that work well in natural hazy outdoor scenes, such as those based on physical models (DCP, Meng Berman) and also those for which the underlying idea of the design is based on physical models (Luzón, IDE). The relatively poor results of IDE may also be related to the fact that the MS-iCID and VIF results are not optimal for this algorithm. Using this algorithm with three-channels images not corresponding to a standard RGB intensity distribution could also have caused a decrease in performance for this method, as well as for Berman to a lesser extent. 

In Galdran’s study [69] AMEF was compared to the DCP, Meng and CLAHE methods and demonstrated a better performance than these methods with less computation time, using a database of synthetic hazy images and other quality metrics (SSIM [83], FADE [79] and CORNIA [88]). This method is probably less conditioned by the fact that the haze in the SHIA database has been artificially generated. 

Regarding the peak position of the optimal bands selected for each algorithm (corresponding to the minimum values of the metrics range shown in Table 1), as expected there is considerable variability in the distribution of peak positions across the algorithms. The method that yields spectral positions that are most similar to the typical RGB peak positions in a conventional camera is the DCP, which is not amongst the best ranked. Nevertheless, DCP provides the widest wavelength range for the triplet selected, with two wavelengths in the red range and one in the blue-green range of the spectrum. The shortest wavelength selected for the DCP (and also by other six dehazing algorithms) is also the shortest in the spectral range of the SHIA database, which is 450 nm. The reduced spectral range is one of the limitations of this database. The CLAHE method has selected bands within the long wavelength range, in which the contrast of the haze-free images is higher, whilst Meng, DehazeNet, Berman, Luzón, IDE and AMEF methods use wavelengths above 450 nm and below 560 nm. The selected triplet by Meng, DehazeNet, Luzón and IDE is the same. In addition, the two shortest wavelengths in the selected triplets by Meng, DehazeNet, Luzón, AMEF and IDE are common (450 nm and 490 nm). This could be related to the fact that relatively poor-quality haze-free and dehazed images tend to be intrinsically more similar, and the quality is low even for the haze-free images near the short end of the spectral range in the SHIA database.

**Figure 2:**
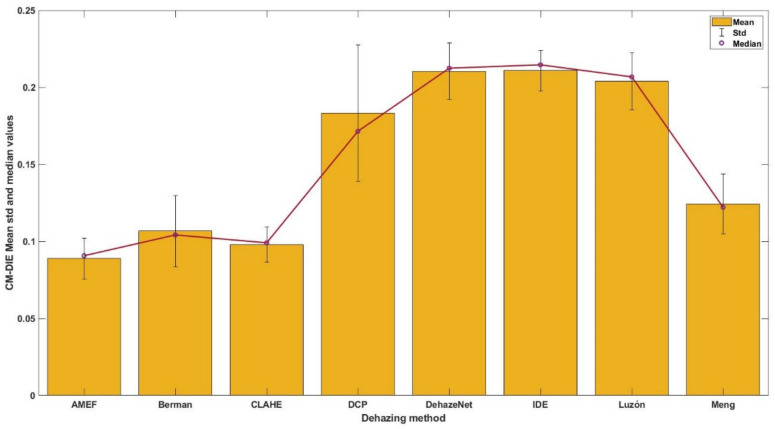
Mean and median of the CM-DIE for each dehazing algorithm in the 1540 band combinations tested. The yellow bars refer to the mean values whilst the blue points represent the median values. The standard deviation values are shown as error bars.

In Figure 2, both the CM-DIE mean and median values for the different algorithms are presented in a bar graph, including the standard deviation as error bars. This figure allows an easier comparison of the performance of the algorithms at a glance. It is clear that the performance of the AMEF, CLAHE, Berman and also the Meng methods is relatively similar. We carried out a statistical analysis of the CM-DIE distributions to elucidate if the differences in performance were significant. As a first step, we determined whether the distributions were normal or not according to the Kolmogorov–Smirnov test [81]. The results indicated that none of the distributions is normal. We then carried out a parametric test (Wilcoxon Signed Rank [89]) to compare the mean values of the distributions pair-wise. The results of this test show that all pairs are significantly different (p_max_ = 5.06 × 10^−21^). Taking into account both the mean and median values, we observe that there is a negligible difference between these values, which indicates that we are not dealing with skewed distributions.

### 6.2. Visualization of The Optimal Triplets

**Figure 3:**
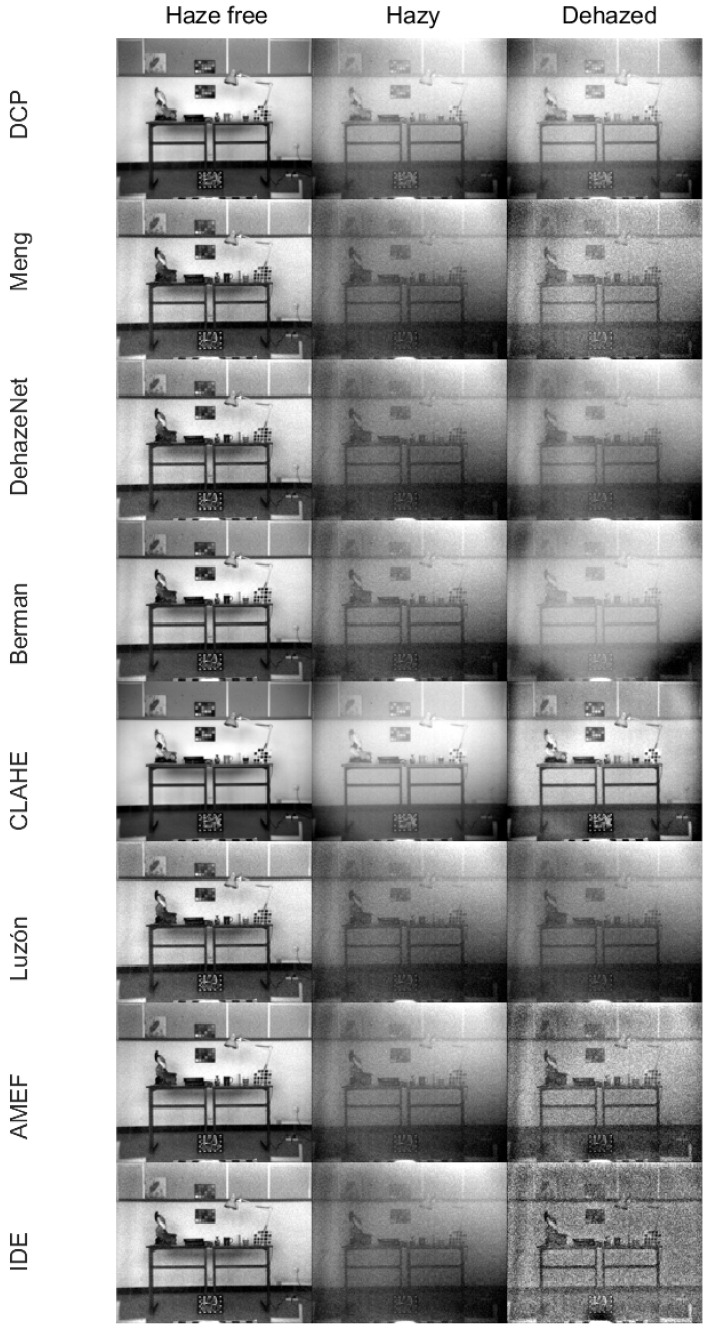
Original haze-free images (**left**), hazy images (next) and dehazed images (**right**). The images correspond to grayscale images generated from the average intensity of the three channels of the optimum triplets for each selected dehazing method, including a contrast stretch step only for visualization purposes.

After the previous quantitative analysis, we concluded that the AMEF [69] is the method that provides the best results. Starting from the optimal triplet for the best value of the combined metric, we generated the grayscale images through the average value of the three channels with each of the methods including a contrast stretch step only for visualization purposes (Figure 3). The left image in each case corresponds to the original haze-free image. Next, the hazy image is shown in the center position. Additionally, thirdly, the image on the right is the dehazed image with each of the methods shown.

The contrast stretch step used in Figure 3 produces images with a high contrast, although they also amplify the presence of some artifacts such as noise. In agreement with the quantitative analysis data, the DehazeNet [66] and Luzón [68] methods show the worst results, removing very little amount of haze. By contrast, IDE [26] seems to have removed a fair amount of haze in spite of the poor sharpness of the dehazed image and the present of notable artifacts. The Meng [65] and Berman [43] methods are in an intermediate performance level, although the Meng method seems to remove a lesser amount of haze; almost all objects can be easily distinguished, but some artifacts were caused by a large number of negative values in the dehazed image, which has been rescaled for display. In the case of the CLAHE [67] and AMEF [69] methods, CLAHE seems to produce visually a better result in the contrast-enhanced grayscale images. This might be linked to the quality of the hazy image corresponding to the optimal bands selected in each case. Since AMEF has selected a triplet of wavelengths within the short-medium range, and CLAHE within the long range, the AMEF optimal triplet image is influenced by the limitations of the database at short wavelengths, already discussed in Section 2. In the case of the optimal triplet, the two algorithms are not dealing with the same haze-free and hazy images, and the hazy image for CLAHE is noticeably less noisy and it contains a lesser amount of haze than AMEF’s hazy image.

Despite having chosen image quality metrics that evaluate different aspects of the retrieved image (Table 1), it is important to emphasize that, as previously mentioned in the literature [85], the existing image quality evaluation metrics do not provide us with information that can precisely reproduce a quality assessment based on the observed visual appearance.

The results for this evaluation were obtained using a PC with an Intel Core i7-8700K 3.7 GHz processor with 64 GB of RAM. The mean (and standard deviation) processing times for 50 runs of each dehazing method with 1312 × 1082 pixels images are shown in Table 2. The slowest method is DCP, and the two fastest methods are Luzón and CLAHE.

### 6.3. sRGB Rendering from The Spectral Image

**Figure 4:**
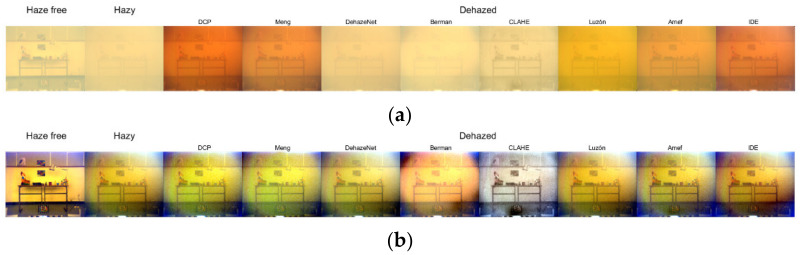
From left to right: Original haze-free image, image with haze and restored images. (**a**) sRGB rendering using the information contained in the 450–720 nm spectral range with a medium haze level (SHIA level 7 [64]); (**a**) images in (**b**) with contrast stretching applied (only for visualization purposes).

Following on from the previous results it is also interesting to analyze the performance of each method through a sRGB rendering from the full spectral images of the SHIA database [64] (see Table 3). In this case, the best result is provided by the AMEF [69] algorithm closely followed by the CLAHE [67] method, according to the CM-DIE metric. The CLAHE method is ranked first for the MS-iCID metric, and the IDE algorithm is ranked first according to MS-SSIM, with a very similar performance to AMEF. The VIF metric ranks DCP as first, and IDE as second best. Overall, the results of IDE have improved considerably when using the sRGB-rendered image, which could indicate that the parameters need to be specifically optimized in this algorithm when adapting it for multispectral or hyperspectral images. In the sRGB rendering, we have information pertaining to the entire visible spectrum, instead of the three bands we had in the generation of our false RGB images analyzed in the previous section. The sRGB-rendered images, as well as a contrast stretched version for visualization purposes, are shown in Figure 4. In this case, a visual analysis of the quality of the dehazed images would agree with the results provided in Table 3 in ranking CLAHE and AMEF as the best-performing methods. This highlights the fact that it is important to compare results using the same image as input for all methods, something which was not possible in Section 6.1 when comparing the optimal triplet images.

The DCP [39] again provides an image with much higher contrast and greater alteration in terms of color, which results in its being ranked first for the VIF metric, but last for the MS-iCiD metric, which is more sensitive to color variations. The same trend is observed for the IDE algorithm. Meng [65] is also ranked third best for VIF and third last for MS-iCID, after DCP and IDE. In addition, we can observe some artifacts in the B channel in the contrast stretched version of the images, that are also present in the hazy image. Something similar can be observed in the case of IDE. Once again, we confirm that DehazeNet [66] and Luzón [68] methods both present a weak haze removal and it is difficult to identify the objects contained in the scene. In CLAHE [67], the color is recovered better than in AMEF [69]. Moreover, for CLAHE the artifacts are much less noticeable than when using the DCP, Meng, Berman and AMEF methods. 

## 7. Conclusions

Our results suggest that algorithms generating an image more similar to the haze-free one and with few artifacts also provide better similarity metrics (AMEF [69], CLAHE [67]). However, the algorithms that tend to generate greater image variations, or that are sensitive to the contrast quality of the original image (DCP [39], CLAHE [67]), tend to perform better with bands around the long wavelengths, which have higher image quality in both the original haze-free and the dehazed images. Not being able to rely on image data with uniform quality across wavelengths within the visible range has been a major limitation in our study.

The optimal wavelengths selected within the restrictions imposed in this study depend on the algorithm used for dehazing, and this fact makes it difficult to obtain a fair visual assessment of the results, even using gray-scale images for displaying the original and dehazed images. It is likely that the optimal wavelengths also depend on the particle size and density.

Methods based on image processing techniques (such as CLAHE [67]) obtain better results than those based on physical methods (e.g., DCP [39]), although this fact might be related to the use of artificially created fog in the database, which might not be as well described by the physical models as naturally occurring haze.

The sRGB rendering of the images obtained with the full spectrum of the scene shows different quality results than the average in the groups of three bands pre-selected before the brute-force optimization. The AMEF algorithm produces better results for the optimal triplet according to the quality metrics used, while CLAHE is ranked as second best for the sRGB image and for the optimal triplet. In general, we can conclude that using the full spectrum does not lead to better results according to the quality metrics and visual appearance of the images (save for the IDE algorithm), and so it would be a much better solution to capture only the information in the optimal bands. This suggests that trying to reduce the complexity of a hyperspectral capture device by using a reduced number of relatively narrow bands is a good strategy. Despite losing a certain amount of radiance, it is very likely that a conventional sensor would be able to deliver enough signal in each of the three bands to allow a high quality final captured image in outdoor conditions.

## Figures and Tables

**Table 1:** Distribution of the quality metrics (mean, standard deviation and range) for the metrics used in this study. The fourth column shows the wavelengths of the optimum bands selected by brute force. The best-performing algorithm is shown in blue, and the second best in green.

Method	Mean Value (std)	Range	Best Value Triplet (nm)	Mean Value (std)			
	CM-DIE		R-G-B	PSNR [81]	MS-SSIM [46]	VIF [47]	MS-iCiD [48]
DCP [39]	0.186 (0.045)	0.015–0.341	710–530–450	14.901 (0.989)	0.856 (0.043)	1.370 (0.106)	0.165 (0.033)
Meng [65]	0.126 (0.020)	0.061–0.174	530–490–450	24.753 (1.346)	0.921 (0.011)	0.752 (0.082)	0.146 (0.030)
DehazeNet [66]	0.216 (0.018)	0.133–0.255	530–490–450	26.017 (1.461)	0.896 (0.021)	0.387 (0.026)	0.207 (0.036)
Berman [43]	0.108 (0.024)	0.059–0.249	560–510–450	25.378 (2.260)	0.924 (0.014)	0.885 (0.136)	0.140 (0.028)
CLAHE [67]	0.098 (0.012)	0.064–0.124	710–650–610	27.515 (1.259)	0.929 (0.003)	1.114 (0.047)	0.137 (0.031)
Luzón [68]	0.209 (0.019)	0.128–0.236	530–490–450	25.632 (1.489)	0.897 (0.021)	0.412 (0.017)	0.201 (0.033)
AMEF [69]	0.089 (0.013)	0.048–0.119	550–490–450	27.086 (1.501)	0.931 (0.010)	0.895 (0.045)	0.115 (0.018)
IDE [26]	0.211 (0.016)	0.137–0.238	530–490–450	24.679 (1.551)	0.894 (0.021)	0.399 (0.014)	0.211 (0.032)

**Table 2:** Mean processing time and standard deviation for 50 runs of each dehazing method with RGB images of 1312 × 1082 pixels for each selected dehazing method.

Method	Mean Runtime (s)	Standard Deviation (s)
DCP [39]	64.684	2.790
Meng [65]	3.621	0.114
DehazeNet [66]	6.506	0.362
Berman [43]	4.169	0.182
CLAHE [67]	0.537	0.034
Luzón [68]	0.083	0.009
AMEF [69]	1.871	0.106
IDE [26]	5.173	0.147

**Table 3:** Quantitative results of sRGB rendering image quality metrics for the different dehazing methods. The best method is marked in bold.

Method	PSNR [81]	CM-DIE	MS-SSIM [46]	VIF [47]	MS-iCiD [48]
DCP [39]	8.638	0.145	0.931	**0.736**	0.224
Meng [65]	11.084	0.134	0.941	0.691	0.183
DehazeNet [66]	25.836	0.191	0.931	0.297	0.165
Berman [43]	26.388	0.202	0.926	0.263	0.173
CLAHE [67]	**27.818**	0.128	0.939	0.651	**0.141**
Luzón [68]	11.761	0.168	0.932	0.450	0.164
AMEF [69]	12.671	**0.127**	0.949	0.632	0.149
IDE [26]	10.553	0.131	**0.951**	0.711	0.200

## Data Availability

Not applicable.

## References

[1] Petty G.W. (2006). A First Course in Atmospheric Radiation.

[2] Liou K.-N. (2002). An Introduction to Atmospheric Radiation.

[3] Gomes A.E., Linhares J.M., Nascimento S.M. (2020). Near perfect visual compensation for atmospheric color distortions. Color Res. Appl..

[4] McCartney E.J. (1976). Optics of the Atmosphere: Scattering by Molecules and Particles.

[5] Martínez-Domingo M.Á., Valero E.M., Nieves J.L., Molina-Fuentes P.J., Romero J., Hernández-Andrés J. (2020). Single Image Dehazing Algorithm Analysis with Hyperspectral Images in the Visible Range. Sensors.

[6] Tarel J.P., Hautiere N., Cord A., Gruyer D., Halmaoui H. Improved visibility of road scene images under heterogeneous fog. Proceedings of the 2010 IEEE Intelligent Vehicles Symposium.

[7] Halmaoui H., Cord A., Hautière N. Contrast restoration of road images taken in foggy weather. Proceedings of the 2011 IEEE International Conference on Computer Vision Workshops (ICCV Workshops).

[8] Mehra A., Mandal M., Narang P., Chamola V. (2020). ReViewNet: A Fast and Resource Optimized Network for Enabling Safe Autonomous Driving in Hazy Weather Conditions. IEEE Trans. Intell. Transp. Syst..

[9] Jia Z., Wang H., Caballero R.E., Xiong Z., Zhao J., Finn A. A two-step approach to see-through bad weather for surveillance video quality enhancement. Proceedings of the 2011 IEEE International Conference on Robotics and Automation.

[10] Cao Z., Qin Y., Jia L., Xie Z., Liu Q., Ma X., Yu C. (2020). Haze Removal of Railway Monitoring Images Using Multi-Scale Residual Network. IEEE Trans. Intell. Transp. Syst..

[11] Hajjami J., Napoléon T., Alfalou A. (2020). Adaptation of Koschmieder dehazing model for underwater marker detection. Pattern Recognition and Tracking XXXI.

[12] Ye D., Yang R. (2020). Gradient Information-Orientated Colour-Line Priori Knowledge for Remote Sensing Images Dehazing. Sens. Imaging Int. J..

[13] Makarau A., Richter R., Muller R., Reinartz P. (2014). Haze Detection and Removal in Remotely Sensed Multispectral Imagery. IEEE Trans. Geosci. Remote. Sens..

[14] Negru M., Nedevschi S., Peter R.I. (2015). Exponential Contrast Restoration in Fog Conditions for Driving Assistance. IEEE Trans. Intell. Transp. Syst..

[15] Hassan H., Bashir A.K., Ahmad M., Menon V.G., Afridi I.U., Nawaz R., Luo B. (2020). Real-time image dehazing by superpixels segmentation and guidance filter. J. Real-Time Image Process..

[16] Cimtay Y. (2021). Smart and real-time image dehazing on mobile devices. J. Real-Time Image Process..

[17] Xie Z., Li Y., Niu J., Shi L., Wang Z., Lu G. (2020). Hyperspectral face recognition based on sparse spectral attention deep neural networks. Opt. Express.

[18] Puzović S., Petrović R., Pavlović M., Stanković S. Enhancement Algorithms for Low-Light and Low-Contrast Images. Proceedings of the 19th International Symposium INFOTEH-JAHORINA (INFOTEH).

[19] Purohit K., Mandal S., Rajagopalan A.N. (2019). Multilevel weighted enhancement for underwater image dehazing. J. Opt. Soc. Am. A.

[20] Wang W., Yuan X. (2017). Recent advances in image dehazing. IEEE/CAA J. Autom. Sin..

[21] Li Y., You S., Brown M.S., Tan R.T. (2017). Haze visibility enhancement: A Survey and quantitative benchmarking. Comput. Vis. Image Underst..

[22] Zhang K., Wu C., Miao J., Yi L. (2013). Research About Using the Retinex-Based Method to Remove the Fog from the Road Traffic Video. ICTIS 2013 Improv. Multimodal Transp. Syst. Inf. Saf. Integr..

[23] Rong Z., Jun W.L. (2014). Improved wavelet transform algorithm for single image dehazing. Optik.

[24] Verma M., Kaushik V.D., Pathak V.K. An efficient deblurring algorithm on foggy images using curvelet transforms. Proceedings of the Third International Symposium on Women in Computing and Informatics.

[25] Ju M., Ding C., Guo Y.J., Zhang D. (2019). IDGCP: Image Dehazing Based on Gamma Correction Prior. IEEE Trans. Image Process..

[26] Ju M., Ding C., Ren W., Yang Y., Zhang D., Guo Y.J. (2021). IDE: Image Dehazing and Exposure Using an Enhanced Atmospheric Scattering Model. IEEE Trans. Image Process..

[27] Feng C., Zhuo S., Zhang X., Shen L., Süsstrunk S. Near-infrared guided color image dehazing. Proceedings of the IEEE International Conference on Image Processing.

[28] Guo F., Zhao X., Tang J., Peng H., Liu L., Zou B. (2020). Single image dehazing based on fusion strategy. Neurocomputing.

[29] Oakley J.P., Satherley B.L. (1998). Improving image quality in poor visibility conditions using a physical model for contrast degradation. IEEE Trans. Image Process..

[30] Zhang X., Jiang R., Wang T., Luo W. (2021). Single Image Dehazing via Dual-Path Recurrent Network. IEEE Trans. Image Process..

[31] Zhang X., Wang T., Wang J., Tang G., Zhao L. (2020). Pyramid Channel-based Feature Attention Network for image dehazing. Comput. Vis. Image Underst..

[32] Deng Z., Zhu L., Hu X., Fu C.-W., Xu X., Zhang Q., Qin J., Heng P.-A. Deep Multi-Model Fusion for Single-Image Dehazing. Proceedings of the IEEE/CVF International Conference on Computer Vision.

[33] Shao Y., Li L., Ren W., Gao C., Sang N. Domain Adaptation for Image Dehazing. Proceedings of the IEEE/CVF Conference on Computer Vision and Pattern Recognition.

[34] Narasimhan S.G., Nayar S.K. Chromatic framework for vision in bad weather. Proceedings of the IEEE Conference on Computer Vision and Pattern Recognition CVPR.

[35] Xu Z., Liu X., Chen X. Fog removal from video sequences using contrast limited adaptive histogram equalization. Proceedings of the International Conference on Computational Intelligence and Software Engineering.

[36] Stark J. (2000). Adaptive image contrast enhancement using generalizations of histogram equalization. IEEE Trans. Image Process..

[37] Joshi K.R., Kamathe R.S. Quantification of retinex in enhancement of weather degraded images. Proceedings of the International Conference on Audio, Language and Image Processing.

[38] Narasimhan S.G., Nayar S.K. (2003). Contrast restoration of weather degraded images. IEEE Trans. Pattern Anal. Mach. Intell..

[39] He K., Sun J., Tang X. (2010). Single image haze removal using dark channel prior. IEEE Trans. Pattern Anal. Mach. Intell..

[40] Wang W., Yuan X., Wu X., Liu Y. (2017). Dehazing for images with large sky region. Neurocomputing.

[41] el Khoury J., le Moan S., Thomas J.-B., Mansouri A. (2018). Color and sharpness assessment of single image dehazing. Multimed. Tools Appl..

[42] Luzón-González R., Nascimento S.M.C., Masuda O., Romero J. (2013). Chromatic losses in natural scenes with viewing distance. Color Res. Appl..

[43] Berman D., Avidan S. Non-local image dehazing. Proceedings of the IEEE Conference on Computer Vision and Pattern Recognition.

[44] Wang Z., Simoncelli E., Bovik A. Multiscale structural similarity for image quality assessment. Proceedings of the The Thrity-Seventh Asilomar Conference on Signals, Systems & Computers.

[45] Sheikh H.R., Bovik A.C. (2006). Image information and visual quality. IEEE Trans. Image Process..

[46] Preiss J., Fernandes F., Urban P. (2014). Color-Image Quality Assessment: From Prediction to Optimization. IEEE Trans. Image Process..

[47] Narasimhan S.G., Wang C., Nayar S.K. (2002). All the images of an outdoor scene. European Conference on Computer Vision.

[48] CAVE | Software: WILD: Weather and Illumination Database. https://www.cs.columbia.edu/CAVE/software/wild/index.php.

[49] el Khoury J., Thomas J.-B., Mansouri A. (2016). A color image database for haze model and dehazing methods evaluation. International Conference on Image and Signal Processing.

[50] Lüthen J., Wörmann J., Kleinsteuber M., Steurer J. (2017). A RGB/NIR Data Set For Evaluating Dehazing Algorithms. Electron. Imaging.

[51] Ma K., Liu W., Wang Z. Perceptual evaluation of single image dehazing algorithms. Proceedings of the IEEE International Conference on Image Processing.

[52] Linhares J., Pinto P.D., Nascimento S.M.C. (2008). The number of discernible colors in natural scenes. J. Opt. Soc. Am. A.

[53] Tarel J.-P., Hautiere N., Caraffa L., Cord A., Halmaoui H., Gruyer D. (2012). Vision Enhancement in Homogeneous and Heterogeneous Fog. IEEE Intell. Transp. Syst. Mag..

[54] Ancuti C., Ancuti C.O., de Vleeschouwer C. D-hazy: A dataset to evaluate quantitatively dehazing algorithms. Proceedings of the IEEE International Conference on Image Processing (ICIP).

[55] Cordts M., Omran M., Ramos S., Rehfeld T., Enzweiler M., Benenson R., Franke U., Roth S., Schiele B. The cityscapes dataset for semantic urban scene understanding. Proceedings of the IEEE Conference on Computer Vision and Pattern Recognition.

[56] Zhang Y., Ding L., Sharma G. HazeRD: An outdoor scene dataset and benchmark for single image dehazing. Proceedings of the IEEE International Conference on Image Processing (ICIP).

[57] Sakaridis C., Dai D., Hecker S., van Gool L. Model adaptation with synthetic and real data for semantic dense foggy scene understanding. Proceedings of the European Conference on Computer Vision (ECCV).

[58] Liu F., Shen C., Lin G., Reid I. (2015). Learning Depth from Single Monocular Images Using Deep Convolutional Neural Fields. IEEE Trans. Pattern Anal. Mach. Intell..

[59] Li B., Ren W., Fu D., Tao D., Feng D., Zeng W., Wang Z. (2018). Benchmarking Single-Image Dehazing and Beyond. IEEE Trans. Image Process..

[60] Ancuti C., Ancuti C.O., Timofte R., de Vleeschouwer C. I-HAZE: A dehazing benchmark with real hazy and haze-free indoor images. Proceedings of the International Conference on Advanced Concepts for Intelligent Vision Systems.

[61] Ancuti C.O., Ancuti C., Timofte R., de Vleeschouwer C. O-haze: A dehazing benchmark with real hazy and haze-free outdoor images. Proceedings of the IEEE Conference on Computer Vision and Pattern Recognition Workshops.

[62] Ancuti C.O., Ancuti C., Sbert M., Timofte R. Dense-haze: A benchmark for image dehazing with dense-haze and haze-free images. Proceedings of the IEEE International Conference on Image Processing (ICIP).

[63] Ancuti C.O., Ancuti C., Timofte R. NH-HAZE: An image dehazing benchmark with non-homogeneous hazy and haze-free images. Proceedings of the IEEE/CVF Conference on Computer Vision and Pattern Recognition Workshops.

[64] el Khoury J., Thomas J.-B., Mansouri A. A Spectral Hazy Image Database. Proceedings of the International Conference on Image and Signal Processing.

[65] Meng G., Wang Y., Duan J., Xiang S., Pan C. Efficient Image Dehazing with Boundary Constraint and Contextual Regularization. Proceedings of the IEEE International Conference on Computer Vision.

[66] Cai B., Xu X., Jia K., Qing C., Tao D. (2016). DehazeNet: An End-to-End System for Single Image Haze Removal. IEEE Trans. Image Process..

[67] Xu Z., Liu X., Ji N. Fog Removal from Color Images using Contrast Limited Adaptive Histogram Equalization. Proceedings of the 2nd International Congress on Image and Signal Processing.

[68] Romero J., Partal D., Nieves J.L., Hernández-Andrés J. Sensor-response-ratio constancy under changes in natural and artificial illuminants. Color Res. Appl..

[69] Galdran A. (2018). Image dehazing by artificial multiple-exposure image fusion. Signal Process..

[70] Bianco S., Celona L., Piccoli F., Schettini R. High-resolution single image dehazing using encoder-decoder architecture. Proceedings of the IEEE/CVF Conference on Computer Vision and Pattern Recognition Workshops.

[71] Bianco S., Celona L., Piccoli F. Single Image Dehazing by Predicting Atmospheric Scattering Parameters. Proceedings of the London Imaging Meeting Society for Imaging Science and Technology.

[72] Pizer S.M., Amburn E.P., Austin J.D., Cromartie R., Geselowitz A., Greer T., Romeny B.T.H., Zimmerman J.B., Zuiderveld K. (1987). Adaptive histogram equalization and its variations. Comput. Vision Graph. Image Process..

[73] Luzón-González R., Nieves J.L., Romero J. (2015). Recovering of weather degraded images based on RGB response ratio constancy. Appl. Opt..

[74] Zhang L., Shen Y., Li H. (2014). VSI: A Visual Saliency-Induced Index for Perceptual Image Quality Assessment. IEEE Trans. Image Process..

[75] Zhang L., Zhang L., Mou X., Zhang D. (2011). FSIM: A feature similarity index for image quality assessment. IEEE Trans. Image Process..

[76] Hautière N., Tarel J.-P., Aubert D., Dumont E. (2008). Blind Contrast Enhancement Assessment by Gradient Ratioing at Visible Edges. Image Anal. Ster..

[77] Fang S., Yang J., Zhan J., Yuan H., Rao R. Image quality assessment on image haze removal. Proceedings of the Chinese Control and Decision Conference (CCDC).

[78] Guo F., Tang J., Cai Z.-X. (2014). Objective measurement for image defogging algorithms. J. Central South Univ..

[79] Choi L.K., You J., Bovik A.C. (2015). Referenceless prediction of perceptual fog density and perceptual image defogging. IEEE Trans. Image Process..

[80] Mittal A., Soundararajan R., Bovik A.C. (2012). Making a “completely blind” image quality analyzer. IEEE Signal Process. Lett..

[81] Johnson D.H. (2006). Signal-to-noise ratio. Scholarpedia.

[82] Grillini F., Thomas J.B., George S. (2021). Comparison of Imaging Models for Spectral Unmixing in Oil Painting. Sensors.

[83] Wang Z., Bovik A.C., Sheikh H.R., Simoncelli E.P. (2004). Image quality assessment: From error visibility to structural similarity. IEEE Trans. Image Process..

[84] Wang Z., Bovik A.C., Lu L. Why is image quality assessment so difficult?. Proceedings of the 2002 IEEE International Conference on Acoustics, Speech, and Signal Processing.

[85] Khoury J. (2016). Model and Quality Assessment of Single Image Dehazing. Ph.D. Thesis.

[86] Lissner I., Preiss J., Urban P., Lichtenauer M.S., Zolliker P. (2012). Image-difference prediction: From grayscale to color. IEEE Trans. Image Process..

[87] Stokes M. (1996). A Standard Default Color Space for the Internet—sRGB. http://www.color.org/contrib/sRGB.html.

[88] Ye P., Kumar J., Kang L., Doermann D. Unsupervised feature learning framework for no-reference image quality assessment. Proceedings of the 2012 IEEE Conference on Computer Vision and Pattern Recognition.

[89] Woolson R.F. (2007). Wilcoxon signed-rank test. Wiley Encyclopedia of Clinical Trials.

